# Oxidative Stress and Cellular Protein Accumulation Are Present in Keratoconus, Macular Corneal Dystrophy, and Fuchs Endothelial Corneal Dystrophy

**DOI:** 10.3390/jcm12134332

**Published:** 2023-06-28

**Authors:** Linda Vottonen, Ali Koskela, Szabolcs Felszeghy, Adam Wylegala, Katarzyna Kryszan, Iswariyaraja Sridevi Gurubaran, Kai Kaarniranta, Edward Wylegala

**Affiliations:** 1 Department of Ophthalmology, Kuopio University Hospital, 70210 Kuopio, Finland; linda.vottonen@pshyvinvointialue.fi (L.V.); ewylegala@sum.edu.pl (K.K.); 2 Department of Ophthalmology, University of Eastern Finland, 70210 Kuopio, Finland; kai.kaarniranta@uef.fi; 3 Institute of Biomedicine, University of Eastern Finland, Yliopistonranta 1, 70210 Kuopio, Finland; szabolcs.felszeghy@uef.fi; 4 Health Promotion and Obesity Management Unit, Department of Pathophysiology, Faculty of Medical Sciences, Medical University of Silesia, 40-055 Katowice, Poland; adam.wylegala@gmail.com; 5 Ophthalmology Department, Railway Hospital, 40-760 Katowice, Poland; katarzynakryszan@wp.pl (K.K.); edward.wylegala@gmail.com (E.W.); 6 Department of Molecular Genetics, Faculty of Biology and Environmental Protection, University of Lodz, Pomorska 141/143, 90-236 Lodz, Poland; 7 Clinical Department of Ophthalmology, II School of Medicine with the Division of Dentistry in Zabrze, Medical University of Silesia, 40-760 Katowice, Poland

**Keywords:** autophagy, Fuchs endothelial corneal dystrophy, keratoconus, macular corneal dystrophy, molecular chaperones, oxidative stress

## Abstract

The aim of the study was to investigate oxidative stress as well as cellular protein accumulation in corneal diseases including keratoconus (KC), macular corneal dystrophy (MCD), and Fuchs endothelial corneal dystrophy (FECD) at their primary affecting sites. Corneal buttons from KC, MCD, and FECD patients, as well as healthy controls, were analyzed immunohistochemically to evaluate the presence of oxidative stress and the function of the proteostasis network. 4-Fydroxynonenal (4-HNE) was used as a marker of oxidative stress, whereas the levels of catalase and heat-shock protein 70 (HSP70) were analyzed to evaluate the response of the antioxidant defense system and molecular chaperones, respectively. Sequestosome 1 (SQSTM1) levels were determined to assess protein aggregation and the functionality of autophagic degradation. Basal epithelial cells of the KC samples showed increased levels of oxidative stress marker 4-HNE and antioxidant enzyme catalase together with elevated levels of HSP70 and accumulation of SQSTM1. Corneal stromal cells and endothelial cells from MCD and FECD samples, respectively, showed similarly increased levels of these markers. All corneal diseases showed the presence of oxidative stress and activation of the molecular chaperone response to sustain protein homeostasis. However, the accumulation of protein aggregates suggests insufficient function of the protective mechanisms to limit the oxidative damage and removal of protein aggregates via autophagy. These results suggest that oxidative stress has a role in KC, MCD, and FECD at the cellular level as a secondary outcome. Thus, antioxidant- and autophagy-targeted therapies could be included as supporting care when treating KC or corneal dystrophies.

## 1. Introduction

The cornea along with the tear film and lens are important for vision due to their role as refractive interfaces in the eye, bending and focusing the light to the retina [1]. The cornea is transparent and mechanically strong, protecting the inner portion of the eye, while being fixed in terms of its ability to focus light [2]. However, it holds most of the focusing power of the eye [1]. The lens has a limited ability to correct the focusing of light passing from the cornea to the focal point at the retina [3]. Therefore, the structure and correct function of the cornea is important for normal vision.

The cornea consists of three cell-containing layers and two acellular layers [4]. The anterior epithelium contains five to seven layers of epithelial cells which regenerate in a process taking approximately 7–10 days [5]. Corneal epithelial stem cells are located at the limbus where newly formed epithelial cells arise. The middle layer of the cornea, i.e., the stroma, separated from the epithelium by the acellular Bowman’s layer, is the thickest layer, comprising approximately 90% of the total corneal thickness [2]. The stroma is mostly extracellular matrix, with collagen type I as its main component along with other fibrils and lamellae providing mechanical strength and shape, maintained by the keratocytes [6]. Keratocytes, found sparingly from the stroma, are quiescent mesenchymal fibroblasts with the ability to re-enter proliferative repair phenotypes as a result of injury. The most posterior layer of the cornea, the endothelium, is a single-cell layer of endothelial cells separated from the stroma by the acellular Descemet’s membrane [2]. The endothelial cells are quiescent, with the cell cycle being arrested at the G1 phase [7]. The main function of the endothelium is to maintain a healthy stroma by regulating stromal hydration and nutrition as the cornea lacks its own blood supply [8]. The nutrients for the endothelium and stromal cells must be derived from the aqueous humor located at the more posterior side.

Due to its important role as a refractive interface, corneal diseases, such as corneal dystrophies and keratoconus, threaten normal vision and affect patients’ quality of life. Corneal dystrophies can be classified as epithelial and subepithelial, epithelial–stromal, stromal, and endothelial dystrophies [9]. However, these dystrophies are not restricted to a certain layer and can also influence acellular layers [4]. Corneal dystrophies are often inherited and noninflammatory conditions without systemic manifestations. Gradual progression is also a common nominator of corneal dystrophies. Despite the clear link between inheritance and disease prevalence, little is known about the pathological and molecular mechanisms involved in disease onset and progression.

Oxidative stress has been linked to keratoconus (KC), macular corneal dystrophy (MCD), and Fuchs endothelial corneal dystrophy (FECD) [10,11]. Other known corneal conditions to which oxidative stress is associated are pterygium, trauma, and chemical injury [12]. KC is a corneal disease affecting the epithelium, Bowman’s layer, and stroma. It usually begins in puberty and is progressive until 30–40 years of age [13]. KC is the most common primary ectasia, which results in irregular astigmatism, myopia, and reduced visual acuity [14]. MCD is a stromal dystrophy in which the deposition of abnormal proteoglycans leads to loss of corneal transparency and decreased vision [15]. The disease usually begins between 10 and 30 years of age and is recognized throughout the world. The prevalence seems to be higher in communities where consanguinity is common. In MCD, there is a mutation in the *CHST6* gene (carbohydrate sulfotransferase). This gene is thought to be important in producing sulfated keratan sulfate. Due to the role of sulfated keratan as an important glycosaminoglycan among the proteoglycans involved in fibril organization, a lack of sulfated keratan leads to the opacity related to the disease [16]. Furthermore, unsulfated keratan tends to precipitate as deposits affecting stromal clarity [15]. FECD is a bilateral dystrophy of the corneal endothelium characterized by the accelerated loss of morphologically and physiologically altered corneal endothelial cells [17]. Although FECD is primarily a disease of the corneal endothelium, there will be secondary changes in all layers of the cornea. There are two types of FECD, an early-onset form (age of onset ~30 years) and a late-onset form (age of onset ~50 years) [18]. The more prevalent late-onset form is usually inherited in an autosomal dominant fashion, and it has variable penetrance and expressivity [19]. There are also changes in the Descemet’s membrane including the accumulation of extracellular matrix and formation of posterior focal excrescences, i.e., guttae. When FECD progresses, a disruption in the corneal pump-leak function occurs, causing corneal edema. In advanced FECD, subepithelial fibrosis can be found. FECD is the leading cause of corneal transplantation [20].

Despite the link to oxidative stress, little is known about the consequences of oxidative stress on the corneal cellular proteome and especially on the proteostasis network (PN) controlling the protein homeostasis. PN is responsible for protein synthesis, quality control, and degradation and its correct function is critical for cellular wellbeing [21]. Oxidative stress is known to cause an increase in protein oxidation creating a need for functional damage control. Heat-shock proteins (HSPs) remodel damaged proteins back to their active conformations as a first line defense against proteotoxic stress, whereas proteasomes and the autophagy pathway degrade proteins and protein aggregates, respectively, to sustain a functional proteome and ameliorate oxidative stress [21,22].

In this study, corneal buttons from KC, MCD, and FECD patients, as well as healthy controls, were analyzed immunohistochemically to detect oxidative stress, as well as examine protein quality control and degradation at their primary affecting sites. Lipid peroxidation marker 4-HNE (4-hydroxynonenal) and the level of antioxidant enzyme catalase were measured to verify the existence of oxidative stress and antioxidant defense system response. HSP70 and sequestosome 1/p62 (SQSTM1) levels were analyzed to determine whether the function of molecular chaperones and accumulation of protein aggregates, respectively, were changed in patient corneas.

## 2. Materials and Methods

### 2.1. Acquisition of Human Samples and Study Approval

This study adhered to the tenets of the Declaration of Helsinki. All aspects of experimental procedures and protocols were approved by the Board of Ethical Committee of the Medical University of Silesia, Katowice, Poland. All subjects were informed that their participation in the research project was voluntary, although participants were unaware of the specific hypothesis and research question. Participants provided their written informed consent for the human material in this study.

### 2.2. Clinical Procedures and Sample Collection

Retrospective series of human corneal buttons were acquired from the Ophthalmology Department, Railway Hospital, Faculty of Medical Sciences in Zabrze, Medical University of Silesia Katowice, Poland. The diagnosis of different corneal dystrophies was made clinically by corneal specialists and confirmed pathohistologically. Controls (healthy corneal buttons) originated from donor corneas that were retrieved according to the European Eye Bank Association guidelines. If healthy corneas were deemed ineligible for transplantation due to systemic contraindications identified after the multiorgan retrieval process, such as the presence of neoplasia in other organs, they were used as control samples.

All participants (Table 1) underwent a thorough ophthalmologic examination including visual acuity, slit-lamp examination, HRT3 in vivo confocal microscopy (Heidelberg Engineering, Heidelberg, Germany), and anterior segment OCT using Optopol Revo NX (Optopol Technology, Zawiercie, Poland) to confirm the diagnosis of the two different corneal dystrophies studied and exclude any other ophthalmic diseases or dystrophies. Patients with keratoconus had Pentacam HR (Oculus, Weltzar, Germany), Casia 2 (Tomey, Nagoya, Japan) tomography, and Corvis (Oculus) conducted to validate the diagnosis. The healthy controls underwent a similar ophthalmologic examination to ensure that they did not have any other eye diseases or dystrophies. Patients with any present or previous cancer, corneal infections, refractive surgery, and serious systemic diseases were excluded. However, patients with well-treated hypertension, well-treated hypercholesterolemia, and well-treated asthma were included. To the best of our knowledge, female patients included in the study were not under contraceptive control.

The samples were mounted in embedding medium (Tissue-Tek OCT; Miles Laboratories, Elkhart, IN, USA), rapidly frozen in propane chilled with liquid nitrogen (−180 °C), and stored at −80 °C until use. Cryoprotected serial cryosections, 5 μm thick, were cut from the embedded buttons with a cryostat (CM3050S; Leica, Heidelberg, Germany).

### 2.3. Immunohistochemistry

After a random selection of slides from each of the individual subjects, primary antibodies were applied in blocking solution (#IHC-101B; Bethyl laboratories, Montgomery, TX, USA) for 30 min at room temperature to prevent the possibility of nonspecific binding of the primary antibodies. The samples were then immunostained with different primary antibodies as follows: in humidity chambers, antibodies were placed onto sections with anti-Hsp70 (Abcam, Cambridge, UK; diluted 1:50), anti-ubiquitin (Dako, Glostrup, Denmark, diluted 1:200), anti-SQSTM1/p62 (Abcam, Cambridge, UK; diluted 1:100), and anti-4-HNE (LS-C68128, LSBio, Seattle, WA, USA). After an overnight incubation at 4 °C, the slides were rinsed in TBS and the secondary Alexa Fluor 488 antibody (Thermo Fisher Scientific, Waltham, MA, USA; diluted 1:500) was added. Samples were incubated at room temperature for 3 h and rinsed with TBS. A fluorescent nuclear marker DAPI (4′,6-diamidino-2-phenylindole dihydrochloride, #D9542; Sigma, St. Louis, MO, USA) diluted 1:10,000 in TBS was added and incubated for 30 min at room temperature. The sections were rinsed with TBS and covered with Mowiol (Sigma Aldrich, St. Louis, MO, USA) mounting media. The control sections originated from the same corneal buttons, and the validity of the sequential staining was then verified with the same protocol as described above except that one of the primary antibodies was omitted. The negative control samples (primary antibody omitted) displayed only a subminimal autofluorescence signal.

### 2.4. Image Acquisition and Analysis

Image acquisition was performed on an Olympus FV1000 (Olympus Life Science, Walthman, MA, USA) laser scanning system. Eight-bit images were captured sequentially from the green, red, and far-red channels on slices using a 40× objective (NA:1.42, Plan Apochromat). The microscope settings were identical for all scans and kept constant during imaging. Representative scans were taken with a Jenoptik ProgRes C5 (Zeiss, Göttingen, Germany) digital camera mounted onto the microscope. Representative high-power micrographs were taken with ZEN black software and processed with Adobe Photoshop. In all imaging procedures, gamma adjustment was performed on the whole image to maintain appropriate contrast. Representative images from human corneas were used for the demonstration of each observation. Light microscopy images of healthy and diseased corneas were recorded from the stained samples before immunofluorescence microscopy to verify the quality of the section (Supplementary Figure S1).

The immunohistochemical results were examined independently from randomly selected corneal sections by three researchers, and no signal was recorded from technical negative controls (TBS instead of primary antibody). The different marker distribution patterns were analyzed semi-quantitatively using ImageJ software (version 1.49) as described below. For quantitation of the different markers, cells of interest were manually designated as regions of interest (ROI). Special care was taken to select representative areas per section from each individual sample to avoid the risk of regional variation of the pathology. To analyze immunohistochemical images, ImageJ software was used to quantify the level of the positive immunoreaction for each marker studied (intensity of B&W pseudo-color) within each ROI. This was achieved by determining the pixel density of each signal and then converting each individual pixel into a numerical value between 0 (no immunohistochemical reaction or signal at background noise level, which was determined to be below 20) and 255 (highest staining intensity).

### 2.5. Statistical Analysis

All immunohistology data analysis was executed using the Mann–Whitney *U* test in SPSS statistics software (ver. 27.0.0.0, SPSS Inc., Chicago, IL, USA). Resulting *p*-values < 0.05 were considered significant. Data are presented as the mean ± standard deviation (SD) using bar plots. The patient characteristics for age were analyzed using the Mann–Whitney *U* test, and sex differences were analyzed using the chi-square test; *p*-values < 0.05 were considered significant.

## 3. Results

### 3.1. Basal Epithelial Cells of the Keratoconus Corneal Buttons Showed Intracellular Accumulation of SQSTM1 and HSP70 Together with Increased Oxidative Stress

**Figure 1:**
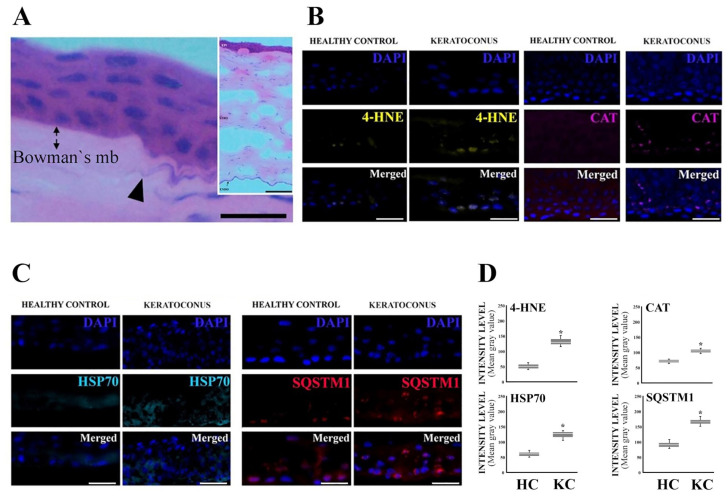
Basal epithelial cells display increased protein aggregation, autophagy, and oxidative stress markers in keratoconus (KC) corneal buttons. (**A**) Hematoxylin/eosin-stained KC cornea showing focal matrix deposit (black arrowhead, Bowman’s mb = Bowman’s layer). (**B**) Representative images of lipid oxidation marker 4-HNE (yellow) and antioxidant enzyme catalase (CAT, purple) immunofluorescense in corneal epithelial cells (DAPI-stained nuclei/blue). (**C**) Representative images of HSP70 (cyan) and SQSTM1 (red) immunofluorescense in corneal epithelial cells (DAPI-stained nuclei/blue). (**D**) Comparative computer-aided densitometric assay of immunostained markers. The gray level intensities of healthy control (HC) and KC corneas represent average intensities. Combined results, *n* = 30. * *p* < 0.001 according to Mann–Whitney *U* test. Results are expressed in box plots as the mean ± SD with whiskers showing minimum and maximum values. The scale bar indicates 100 µm (**A**) or 1 µm (**B**,**C**).

The level of oxidative stress and antioxidant defense system activation were elevated in the corneal epithelial cells of KC patients. Lipid oxidation marker 4-HNE level was almost threefold in comparison to healthy control, and the level of antioxidant enzyme catalase was almost doubled in KC patients (Figure 1). Similarly, level of HSP70 responsible for protein remodeling and SQSTM1 as a marker of accumulation of protein aggregates destined for autophagic degradation showed a clear increase in the epithelial cells of KC patients. Furthermore, intense focal matrix deposit accumulation was observed. KC seems to evoke high levels of oxidative stress, resulting in the activation of protective systems including the antioxidant defense system and protective pathways of PN. However, the accumulation of protein aggregates according to the SQSTM1 signal suggests that the protection is insufficient, leading to accumulation of waste material destined for degradation.

### 3.2. MCD Samples Showed an Increase in Oxidative Stress and Activation of PN Regulatory Pathways in Stromal Cells Together with Presence of Stromal Deposits

**Figure 2:**
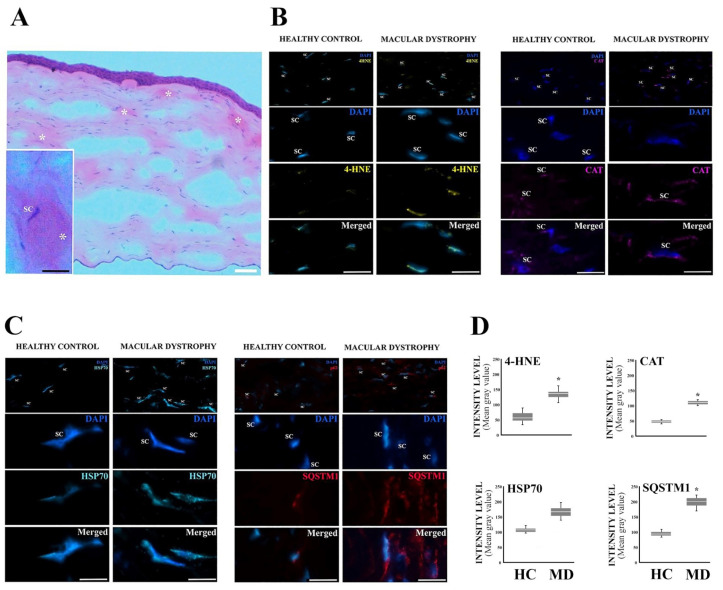
Corneal stromal cells display increased protein aggregation, autophagy, and oxidative stress markers in MCD corneal buttons. (**A**) Hematoxylin/eosin-stained MCD cornea showing stromal deposits (asterisks). SC indicates stromal cell. (**B**) Representative images of 4-HNE (yellow) and catalase (CAT, purple) immunofluorescences in corneal stromal cells (DAPI-stained nuclei/blue) of MCD and healthy control samples. SC indicates stromal cell. (**C**) Representative images of HSP70 (cyan) and SQSTM1 (red) immunofluorescences in corneal stromal cells (DAPI-stained nuclei/blue) of MCD and healthy control samples. SC indicates stromal cell. (**D**) Comparative computer-aided densitometric assay of immunostained markers. The gray level intensities of healthy control (HC) and MCD corneas represent average intensities. Combined results, *n* = 30. * *p* < 0.001 according to Mann–Whitney *U* test. Results are expressed in box plots as mean ± SD with whiskers showing minimum and maximum values. The scale bar indicates 100 µm (**A**) or 1 µm (**B**,**C**).

Compared to controls, cross-sectioned corneas with MCD showed a significantly higher percentage of stromal cells that were immunoreactive for SQSTM1 (red), HSP70 (cyan), and 4-HNE (yellow) (Figure 2). Furthermore, the level of antioxidant enzyme catalase was significantly elevated in the stromal cells of MCD patient corneas. Therefore, oxidative stress and protein damage seem to be a part of the cellular pathology of MCD, evoking antioxidant defense system and PN regulating pathways such as the function of molecular chaperones and autophagic cargo assembly. The light microscopy imaging also revealed presence of stromal deposits associated with the disease pathology.

### 3.3. Oxidative Stress- and Protein Homeostasis-Related Markers Were Increased in Endothelial Cells of FECD Patients’ Samples Together with Cell Elongation

**Figure 3:**
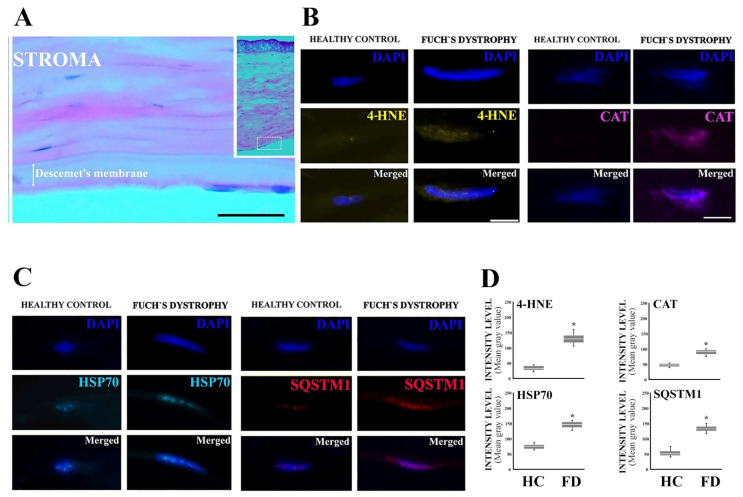
Corneal endothelial cells display increased protein aggregation, autophagy, and oxidative stress markers in FECD corneal buttons. (**A**) Hematoxylin/eosin staining for FECD. (**B**) Representative images of 4-HNE (yellow) and catalase (CAT, purple) immunofluorescences in corneal endothelial cells (DAPI-stained nuclei/blue) of FECD and healthy control samples. (**C**) Representative images of HSP70 (cyan) and SQSTM1 (red) immunofluorescences in corneal endothelial cells (DAPI-stained nuclei/blue) of FECD and healthy control samples. (**D**) Comparative computer-aided densitometric assay of immunostained markers. The gray level intensities of healthy control (HC) and FECD corneas represent average intensities. Combined results, *n* = 30. * *p* < 0.001 according to Mann–Whitney *U* test. Results are expressed in box plots as the mean ± SD with whiskers showing minimum and maximum values. The scale bar indicates 100 µm (**A**) or 1 µm (**B**,**C**).

The endothelial cell layer analyzed from patients with FECD revealed cell elongation together with changes in oxidative stress and protein degradation markers (Figure 3). The levels of 4-HNE and catalase were significantly increased in the patients’ samples compared to healthy ones, suggesting existence of oxidative stress in the endothelial cells. At the same time, HSP70 and SQSTM1 levels increased, pointing to disturbed proteostasis where molecular chaperones try to reassemble damaged proteins. However, the accumulation of autophagy substrate marker SQSTM1 reveals unsuccessful function of chaperones to maintain homeostasis and the proteins begin to accumulate into aggregates destined for autophagic clearance. Moreover, the accumulation of SQSTM1 points to an insufficient removal of protein aggregates via the autophagic pathway.

## 4. Discussion

Oxidative stress has been connected to several corneal diseases including KC, MCD, and FECD [10,11]. Furthermore, there is a linkage association between corneal diseases suggesting potential role for oxidative stress and antioxidant defense system functionality to contribute the wellbeing of the entire cornea along the genetic factors [23,24,25]. Oxidative stress damages many cellular biomolecules, leading to an accumulation of dysfunctional proteins and cell organelles. These typically further induce oxidative stress, creating a vicious cycle. The damaged proteins tend to form larger deposits, passively or actively, which can only be removed via autophagic degradation, stressing its importance to cellular wellbeing. Despite the knowledge of the involvement of oxidative stress in many corneal diseases, the functionality of stress control, including the function of PN at their primary affecting side, may open new opportunities for treatment options. Therefore, the understanding of the function of molecular chaperones remodeling damaged proteins and autophagy in patient material becomes of interest.

Indeed, all the corneal diseases studied here showed increased levels of lipid peroxidation seen as elevated 4-HNE levels. The average intensity levels of 4-HNE were increased approximately threefold in all diseases. Simultaneously, the levels of antioxidant enzyme catalase were increased, possibly as a counteraction against oxidative stress to control oxidative damage. Interestingly, the level of catalase was increased in corneal epithelial cells of KC only by approximately 1.5-fold whereas its expression in corneal stromal cells (MCD) and in endothelial cells (FECD) were more than doubled. Catalase is known to be controlled by transcription factor NFE2L2 (nuclear factor erythroid 2-related factor 2), and its functionality seems to be compromised in KC [26,27,28]. In fact, antioxidant enzymes controlled by other transcription factors have not shown differences in their expression levels between healthy and KC-affected corneas [29]. Furthermore, a meta-analysis showed clear involvement of oxidative stress and antioxidant markers in KC measured from several sample types [30]. However, catalase levels seemed to vary between over- and underexpression among the studies, possibly due to heterogeneity in studied samples.

FECD is also connected to oxidative stress and imbalance in the response against oxidative damage [11,31]. Similarly, the functionality of NFE2L2 and its regulated antioxidative enzyme expression has been connected to the cellular pathogenesis of FECD [32,33]. However, catalase level has not been shown to change and the expression levels of antioxidant genes are rather downregulated than upregulated in FECD [32,34]. Catalase expression is controlled by several antioxidant defense system-related transcription factors, not only by NFE2L2, and the dysfunction of NFE2L2 signaling may be compensated for by other transcription factors, leading to the increase in catalase expression seen in this study [27].

The involvement of oxidative stress or antioxidant defense system in MCD has not been reported before. However, the link between oxidative stress or catalase to granular macular dystrophy has been established [35,36]. Nonetheless, the link between corneal stromal dystrophies and oxidative stress seems to lack evidence. Interestingly, in this study, the MCD corneal stromal cells showed a similar increase in 4-HNE level as seen in KC and FECD with established association to oxidative stress. Furthermore, similarly to other diseases, the level of catalase in MCD patients’ corneal stromal cells was significantly increased, suggesting a role for oxidative stress in the cellular pathogenesis.

However, it should be noted that other players than catalase in the antioxidant defense system or disease-associated oxidative stress may play a role in disease prevalence or progression. For example, in KC corneas, there is a reduced amount of antioxidant enzymes including aldehyde dehydrogenase class 3 (ALDH3) and superoxide dismutase (SOD) necessary to remove the reactive oxygen species (ROS) that increase or accelerate the degenerative process [37]. Furthermore, studies have shown that there is a higher prevalence of KC in countries with hotter climate compared to Europe and North America; there is belief that the high sun exposure in these countries accounts for the higher through ultraviolet (UV)-mediated oxidative stress [37]. Nevertheless, it is likely that the oxidative damage caused by UV radiation combined with the genetic factors such as consanguinity precipitates or accelerates the disease process.

Due to increased oxidative stress, oxidative damage to cellular components and activation of protective pathways are expected. Molecular chaperones play a role in the first line protection against protein damage by remodeling the proteins to their native conformation or guarding their efficient degradation [38]. The HSP70 levels were found to be significantly increased in all corneal diseases in this study. The level of HSP70 was approximately doubled in FECD and KC, whereas a more modest increase was seen in the corneal stromal cells of MCD samples. If the molecular chaperones fail to remodel or secure protein degradation via proteasomes, which is where approximately 80–90% of all proteins are degraded in mammalian cells, damaged poly-ubiquitinated proteins tend to aggregate directly or with assistance by the cell’s protective mechanisms to prevent the spread of oxidative injury [39,40,41]. The ubiquitination of proteins in aggregates is recognized by SQSTM1, a protein that links protein aggregates to the autophagic degradation pathway [42]. After SQSTM1 linkage, autophagic machinery recognizes the cargo for degradation and builds an isolation membrane around the cargo via SQSTM1–LC3B interaction [43]. Finally, the formed autophagosome fuses with a lysosome, and the cargo is degraded along with the protein aggregation marker SQSTM1 [44]. While being a marker for protein aggregation, autophagy machinery constantly builds new autophagosomes and degrades material destined to this pathway, resulting in a decrease in SQSTM1 when activated [44,45]. Therefore, a significant increase in SQSTM1 points to an inadequate removal of protein aggregates and inefficient autophagy.

Elevated SQSTM1 levels were observed in all corneal diseases in this study, suggesting the formation of protein aggregates and defective or inadequate autophagic degradation. Furthermore, SQSTM1 plays a role in multiple subclasses of selective autophagy, such as mitophagy and lipophagy, and the increased SQSTM1 levels may also reflect inefficient removal of cellular organelles resulting in a disturbance in cellular homeostasis [46]. Interestingly, SQSTM1 levels were doubled in MCD and FEDC, whereas, in KC samples, there was roughly a 1.5-fold increase. The levels of SQSTM1 seemed to follow the levels of catalase. This trend may be explained by the function of NFE2L2. Both genes, *catalase* and *SQSTM1*, have a binding site for NFE2L2 and are, therefore, regulated by the same transcription factor [47]. Moreover, corneal epithelial cells are constantly renewed, which may explain the lower accumulation of SQSTM1. The corneal endothelial and stromal cells are quiescent, exposing these cell types to higher oxidative stress and dysfunctional repair mechanisms over time. This may result in a higher accumulation of oxidative damage and SQSTM1-tagged protein aggregates. It should also be noted that the average age of the FECD group was significantly higher compared to healthy controls in this study (Table 1). Furthermore, the FECD group had mostly females, whereas all the control samples were taken from male participants who are known to be more sensitive to oxidative stress [48,49]. Indeed, the function of autophagy, especially mitophagy, in FECD has been shown to change in these post-mitotic cells upon disease development [50,51,52,53]. Furthermore, impairment of the autophagy–lysosomal pathway has been postulated to participate in the accumulation of unsulphated keratan in corneal stroma upon MCD [51]. The changed expression pattern of the studied markers in KC suggests genetic predisposition due to rapid renewal of the epithelial cells, where time may not be a significant factor in damage accumulation. Indeed, ancestry has been shown to play a role in the NFE2L2-mediated antioxidant defense system’s response in KC [28].

However, this study had its limitations. The number of samples, especially in the control group, limited the power of the study. Furthermore, as mentioned before, the average age of the FECD group was significantly higher compared to the control group, which may affect the oxidative stress levels and autophagy functionality. Similarly, the sex imbalance in and between the groups, especially in the control group, may have interfered with the results due to differences between sexes. Moreover, causality could not be estimated from the materials; hence, the relationship of oxidative stress and its consequences to the disease state, for example, could not be assessed. The use of a singular study method for analysis, i.e., immunohistochemistry, can be considered a limiting factor when quantitating protein levels. However, immunohistochemistry provides a method where possible changes can be detected in larger tissue samples at a cellular level without needing extraction methods with possible contamination from adjacent tissues/cells.

**Figure 4:**
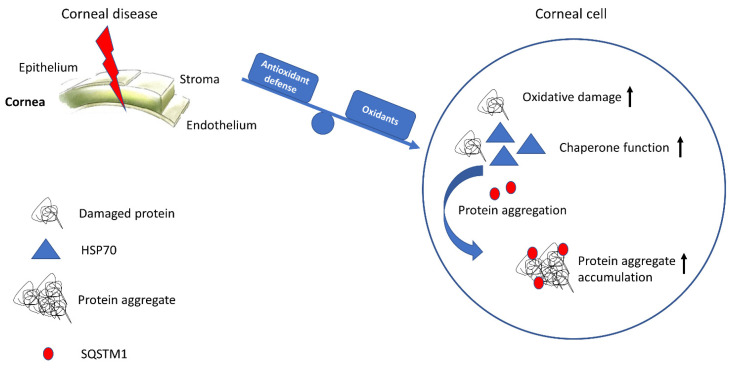
Study summary. Corneal diseases increased oxidative stress in the corneal cells leading to protein damage, increased levels of molecular chaperones controlling the protein damage, and buildup of protein aggregates due to insufficient chaperone function and autophagic degradation.

All corneal diseases inspected, KC, MCD, and FECD, showed increased oxidative damage, antioxidant response, elevated levels of molecular chaperones to limit the oxidative damage, and accumulation of protein aggregate marker SQSTM1 at their primary affecting sites, suggesting inadequate removal of protein aggregates or cellular organelles via autophagy (Figure 4). This creates a state where accumulating oxidative damage feeds the vicious cycle, worsening the situation at every turn. Similar findings have been seen in many aging-related diseases, including Alzheimer’s disease and age-related macular degeneration, where oxidative stress and the accumulation of protein waste due to dysfunctional antioxidant defense system and autophagy are key elements of the cellular pathologies [54,55,56,57,58,59]. However, efficient antioxidant defense system- or autophagy-targeted therapies for these diseases often requires systemic administration and distribution to the tissues known to be well protected by the blood–brain barrier or blood–retinal barrier that select what molecules may pass through [60,61]. The location of the cornea at the anterior side of the eye with exposed cell layers offers an opportunity to treat oxidative stress and its consequences with topical medication, avoiding many barriers set by other administration routes [62]. Antioxidant therapies have been studied in several corneal disease models and clinical studies with encouraging results in oxidative stress and inflammation control [63,64,65,66,67]. However, the antioxidant defense system is regulated by various transcription factors requiring careful planning of the potential therapies. For example, NFE2L2 controls catalase and ALDH3, whereas FOXO (forkhead box transcription factor of the class O) and NF-κB (nuclear factor kappa B) regulate the expression of SOD enzymes [27]. Recently, pinosylvin, an analog of resveratrol, was found to activate both catalase and SOD1 in a mouse model of age-related macular degeneration possibly due to the dual effect of pinosylvin and its metabolite resveratrol on NFE2L2 and SOD regulating transcription factors, respectively [68]. Pinosylvin or a combination of molecules capable of activating the antioxidant defense system in a wide spectrum would possibly offer a higher impact on cellular wellbeing than molecules targeted to a single transcription factor. Furthermore, autophagy-targeted treatments for corneal diseases have also been studied with promising results [69,70]. Trehalose, a sugar molecule, has been shown to not only activate autophagy but also protect cells from UV-induced photoaging by activating the antioxidant defense system including the SOD and GSH/GSSG (glutathione/oxidized glutathione) response [71]. Therefore, despite not being the primary causes of the disease pathology, oxidative stress- and autophagy-targeted topical therapies for KC, MCD, and FECD seem to be promising avenues for new innovations in controlling the spread of oxidative damage and possibly limiting disease progression that could otherwise require corneal surgery and transplantation. However, more in-depth research on the possible advantages of these therapies is needed.

## Figures and Tables

**Table 1:** Patient characteristics.

	*n*	Age (Years, Mean)	*p*	Sex Male/Female	*p*
Control	3	35.3		3/0	
KC	12	39.1	0.613	12/0	NA
MCD	5	39.8	0.549	1/4	0.028
FECD	14	73.3	0.008	3/11	0.010

KC = keratoconus, MCD = macular corneal dystrophy, FECD = Fuchs endothelial corneal dystrophy, *p* = *p*-value, NA = not applicable (all were male). The Mann–Whitney *U* test was used for the calculation of the significance of the difference between age groups (disease vs. control), whereas the chi-square test was used for sex.

## Data Availability

The raw data will be made available by the authors under reasonable request.

## Supplementary Materials

The following supporting information can be downloaded at https://www.mdpi.com/article/10.3390/jcm12134332/s1, Figure S1. Light microscopy images of healthy and diseased corneas from immunostained samples. The scale bar indicates 100 µM.
