# Supplementary material for: Oxidative Stress and Cellular Protein Accumulation Are Present in Keratoconus, Macular Corneal Dystrophy, and Fuchs Endothelial Corneal Dystrophy
Source: J Clin Med. 2023 Jun 28;12(13):4332. doi: 10.3390/jcm12134332 (PMC10342758; doi:10.3390/jcm12134332)
Supplement: Supplementary file 1 [file jcm-12-04332-s001.zip › jcm-2389296-supplementary.pdf]

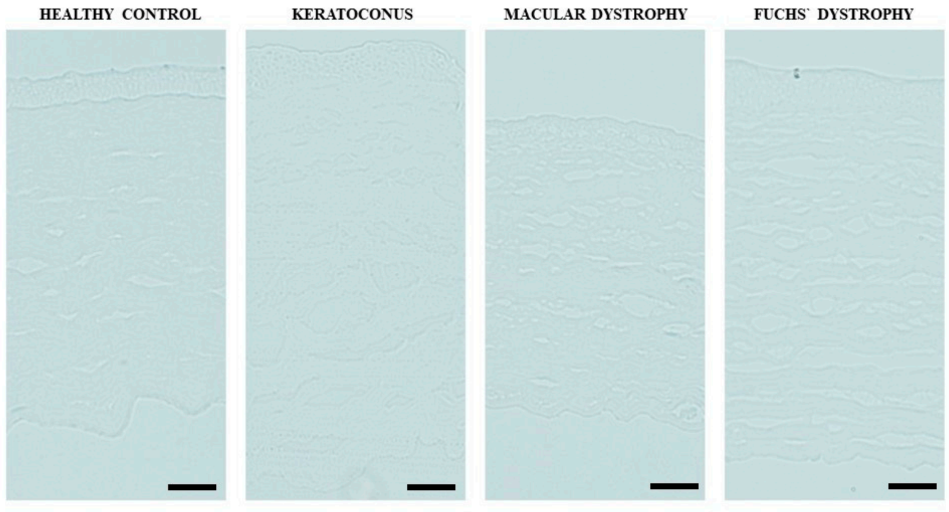

Supplementary Figure S1. Light microscopy images of healthy and diseased corneas from immunostained samples. The scale bar indicates 100 µM.
